# Endoscopy-MR Image Fusion for Image Guided Procedures

**DOI:** 10.1155/2013/472971

**Published:** 2013-11-02

**Authors:** Anwar Abdalbari, Xishi Huang, Jing Ren

**Affiliations:** ^1^Faculty of Engineering and Applied Science, University of Ontario Institute of Technology, 2000 Simcoe Street North, Oshawa, ON, Canada L1H 7K4; ^2^Department of Medical Imaging, University of Toronto, Toronto, ON, Canada M5T 1W7

## Abstract

Minimally invasive endoscope based abdominal procedures provide potential advantages over conventional open surgery such as reduced trauma, shorter hospital stay, and quick recovery. One major limitation of using this technique is the narrow view of the endoscope and the lack of proper 3D context of the surgical site. In this paper, we propose a rapid and accurate method to align intraoperative stereo endoscopic images of the surgical site with preoperative Magnetic Resonance (MR) images. Gridline light pattern is projected on the surgical site to facilitate the registration. The purpose of this surface-based registration is to provide 3D context of the surgical site to the endoscopic view. We have validated the proposed method on a liver phantom and achieved the surface registration error of 0.76 ± 0.11 mm.

## 1. Introduction

In this paper, we develop a new method for endoscopy-MR image fusion of the liver organ for minimally invasive endoscope based surgery. Image guidance is an essential tool in minimally invasive endoscope based abdominal procedures [1]. Effective image guidance can compensate the restricted perception during the operation, which is considered a major limitation in endoscopic procedures. Without image guidance, the surgeon cannot see through the surface of the operation site and may accidentally cause damages to the critical structures of the patient. A typical procedure in image guidance is to map pre-operative high quality MR images to intra-operative endoscopic video images, or the patient thereby provides a good quality context to the real-time endoscopic images. Thus, the surgeon will be able to visually access the operation site during the procedure. As a result, the damage to the critical organs or tissues will be substantially minimized.

Fusion of endoscopic video images with high quality MR images requires good match of these two modalities. In this paper, we adopt a surface based image fusion because the two modalities are different in acquisition and nature [2, 3]. In order to find the corresponding 3D surface model from endoscopic images, we utilize stereovision to snapshot the surgical site from two different angles and compute the 3D location by using triangulation [4]. Cameras are calibrated before triangulation is used [5, 6]. 

Although a liver phantom is used to validate the proposed technique, our method is not restricted to the liver surgery. The integrated image guidance can also be applied to other endoscopic procedures. This paper is organized as follows.  Section 2 introduces the experimental setup and the camera calibration of the stereo endoscope.  Section 3 discusses automatic surface reconstruction, and Section 4 presents surface based registration and experimental results of image fusion.  Section 5 discusses the issues in this study.  Section 6 presents the conclusion and future work.

## 2. Experimental Setup and Camera Calibration

**Figure 1:**
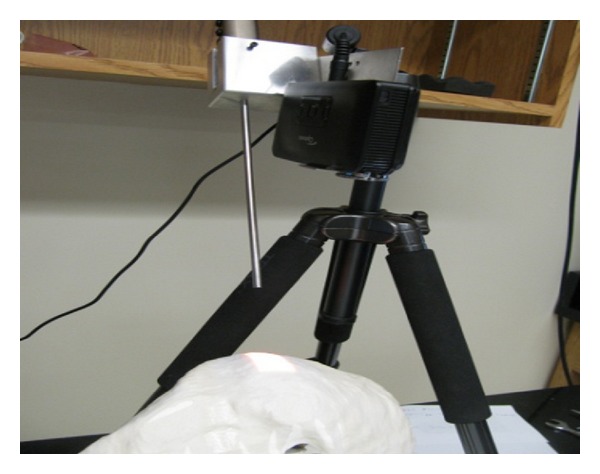
Experimental setup for stereo endoscope and liver phantom.

Experimental setup is shown in Figure 1. In the experiments of this study, we use the following major components: a Visionsense VSII stereo endoscope, an Optoma PK301 Pocket Projector, a liver phantom, and a chessboard calibration pattern. Optoma PK301 Pocket Projector is a small size projector and can be easily mounted. The resolution of this projector is 848 by 480. The liver phantom was printed using a 3D printer based on the liver model that was segmented from MR images of a human subject. 

In this study, robust 3D surface reconstruction requires accurate camera calibration of the stereo endoscope. The calibration process aims to find intrinsic parameters and correct the optical distortion inherent in the endoscope and to compute extrinsic parameters to capture the spatial relationship between left and right cameras of the stereo endoscope. We have modified the Camera Calibration Toolbox for MATLAB [7] and performed calibration of the stereo endoscope using a chessboard calibration pattern.

## 3. Surface Reconstruction from Stereo Endoscope

**Figure 2:**
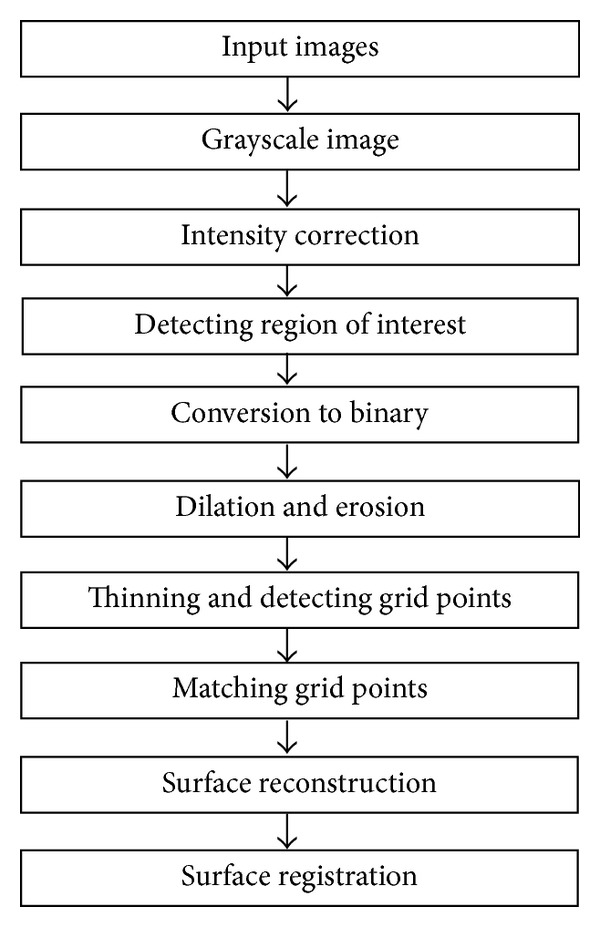
Flowchart for automatic fusion of intraoperative endoscopic images to preoperative images.

In this section, we propose a novel approach to reconstruct the surface of the surgical site from two stereo endoscopic images. The reconstruction procedure is shown in Figure 2. First, a gridline pattern is projected on the surgical site, and both left and right images are acquired at the same time. Second, the intersection points of the gridlines are automatically detected and matched in both images. Then we reconstruct the surface with the matched intersection points. We will describe major steps in detail in the following sections.

### 3.1. Conversion of Input Images to Grayscale Images

**Figure 3:**
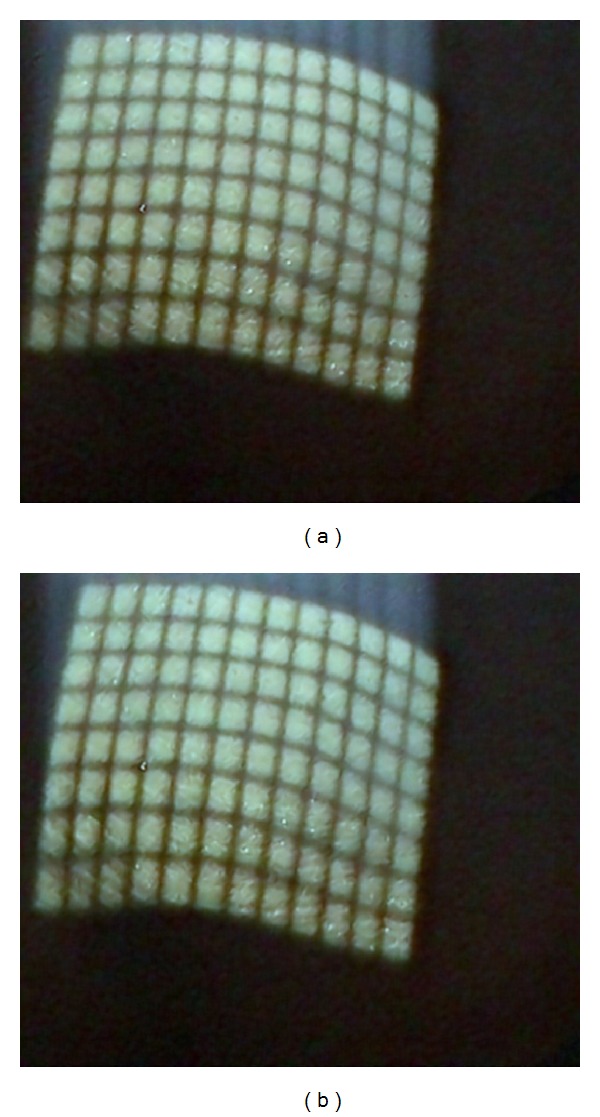
Original stereo endoscopic color images: (a) left image and (b) right image.

**Figure 4:**
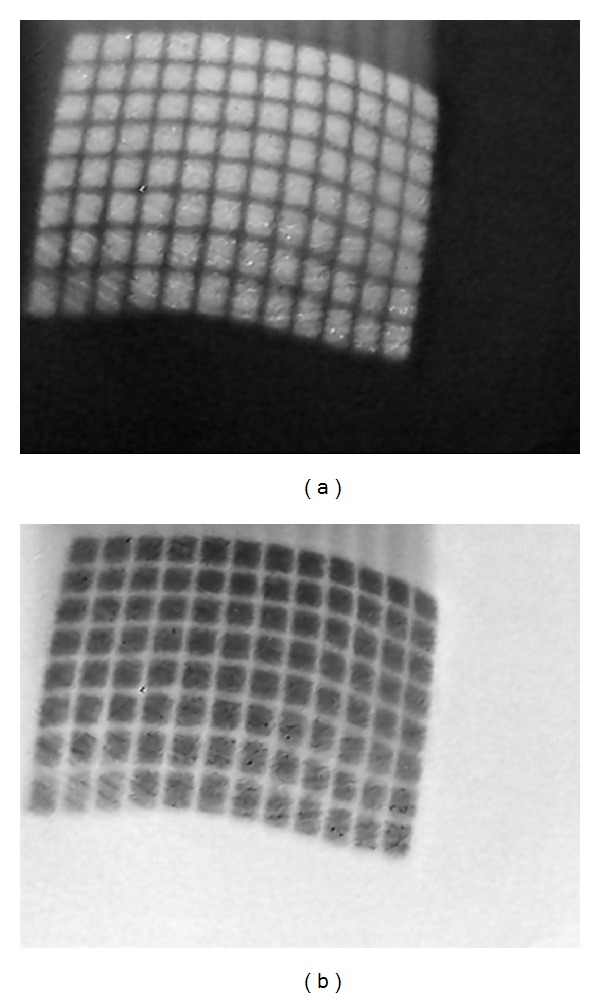
Left (a) grayscale image and (b) negative image.

In order to detect the intersections of the grid lines pattern of an image, we use the image of binary format as algorithm input. We first convert the color images acquired from the stereo endoscope to grayscale images (as shown in Figures 3 and 4). According to the thinning algorithm used in the proposed system, the gridlines of the light pattern should be bright to detect their intersections. The grayscale image is thus inverted to meet this constraint. In this process, the dark areas become bright and vice versa. Next, multiple steps are employed to obtain good binary images.

### 3.2. Intensity Correction

The image intensity of the endoscopic images is not uniform given that variation in illumination and ambient lights exist. As a consequence, conventional threshold methods cannot be directly used to achieve good binary images which can successfully separates gridlines from the background. In this paper, we present an intensity correction technique to improve the image. The improvement aims to equalize the contrast between gridlines and background over the whole image. The new corrected pixel value is calculated by

(1) $$\begin{array}{c} I_{\text{new}}=255-\left(\frac{\left(I_{\text{ave}}-I_{c}\right)}{2}+127\right), \end{array}$$

where *I*_*c*_ is the intensity value of the current pixel, *I*_ave_ is the avarage intensity of its neighbourhood pixels, and *I*_new_ is the new intensity value after correction.

**Figure 5:**
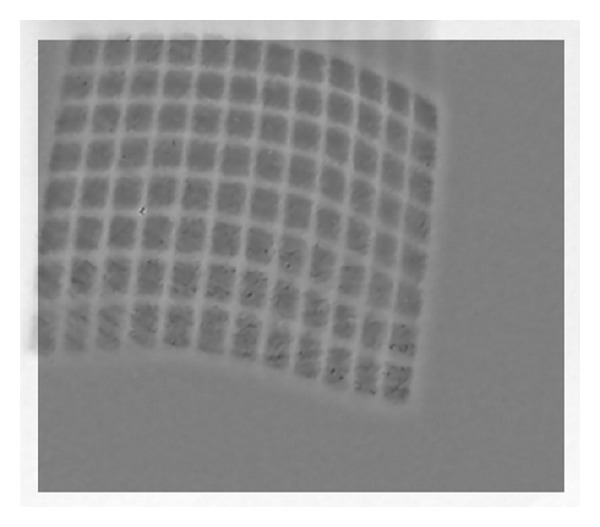
Intensity correction image.

**Figure 6:**
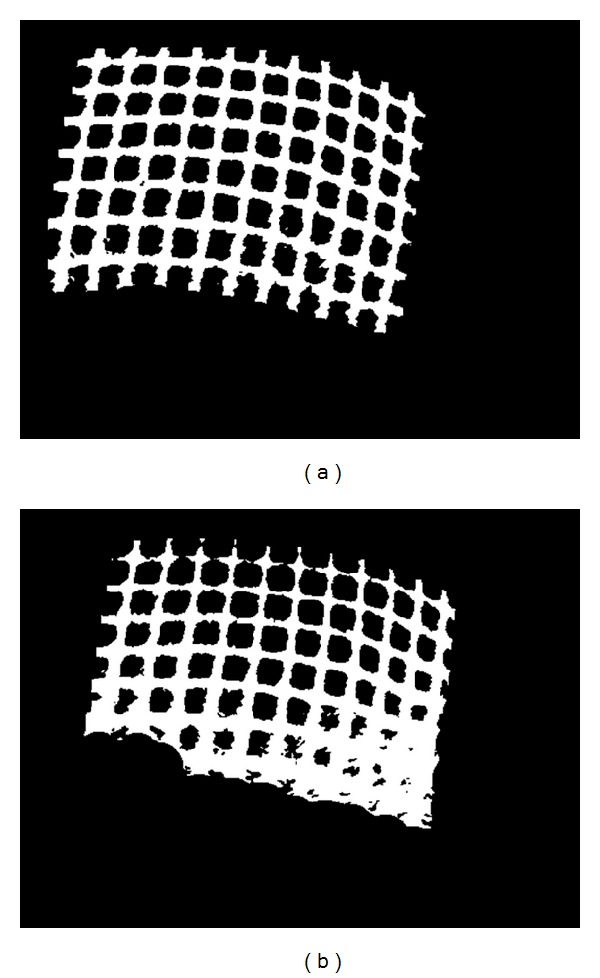
Binary image by thresholding (a) with intensity correction and (b) without intensity correction.

Figure 5 shows the image after intensity correction, in which the contrast between the gridlines and background is more uniform compared with the image before correction in Figure 4(b).  Figure 6 shows binary images by thresholding, which will be used for intersection detection. With intensity correction, all gridlines are clearly shown in the binary image, while without intensity correction, only a part of the gridlines is shown in the cluttered binary image. The intensity correction also significantly improves the detection and matching accuracy with the successful rate of 98% versus 57% without intensity correction (see Table 1).

### 3.3. Detection of Region of Interest

In this paper, region of interest (ROI) is defined as the region which only covers the projected gridline light pattern in the endoscopic image. Automatic detection of ROI is critical for accurate detection and matching of intersection points in the gridline pattern. During the image preprocessing step, the area out of ROI should be cut out. ROI detection leads to automatic removal of unwanted areas. This step significantly improves the correctness of gridline intersection detection as well as the processing speed.

**Figure 7:**
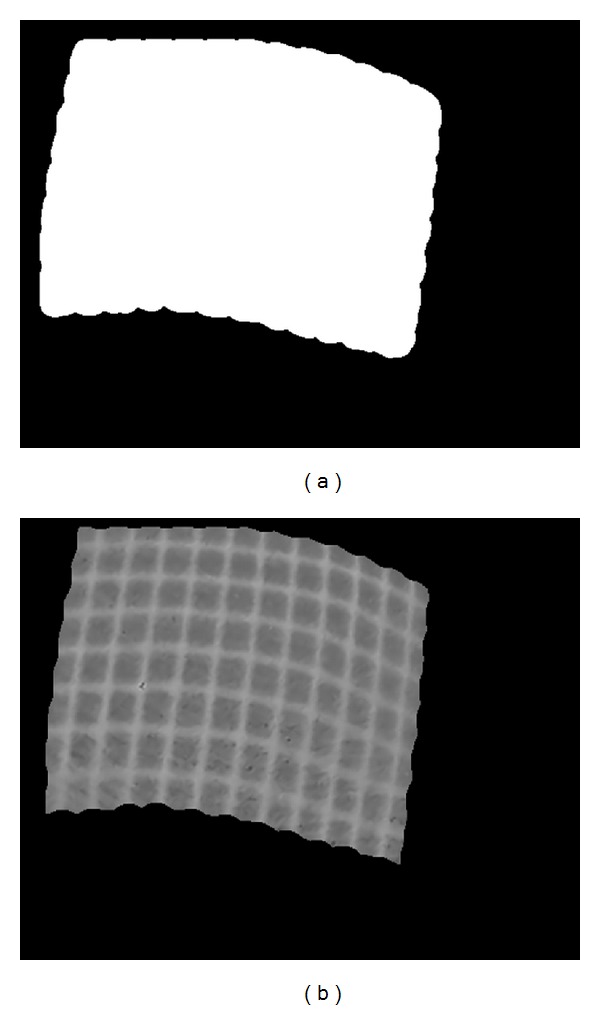
(a) Detected ROI mask image and (b) intensity correction image within ROI mask.

 The ROI detection process aims to generate a mask of the grid lines pattern. Following intensity correction, we threshold the images in order to convert the corrected grayscale image into a binary image for further processing. Next, the dilation and the erosion operations are performed. Eventually, as the consequence of dilation and erosion processes, we obtain a binary mask image only covering the region of the projected gridline pattern. Then, we apply the mask image to the intensity corrected image to produce a cropped image within the desired ROI. The cropped image is then converted to a binary image by applying a threshold to it, which is used for feature detection.  Figure 7(a) shows the detected ROI of the input image (ROI mask image), and Figure 7(b) shows the cropped image within ROI. 

### 3.4. Image Dilation and Erosion

**Figure 8:**
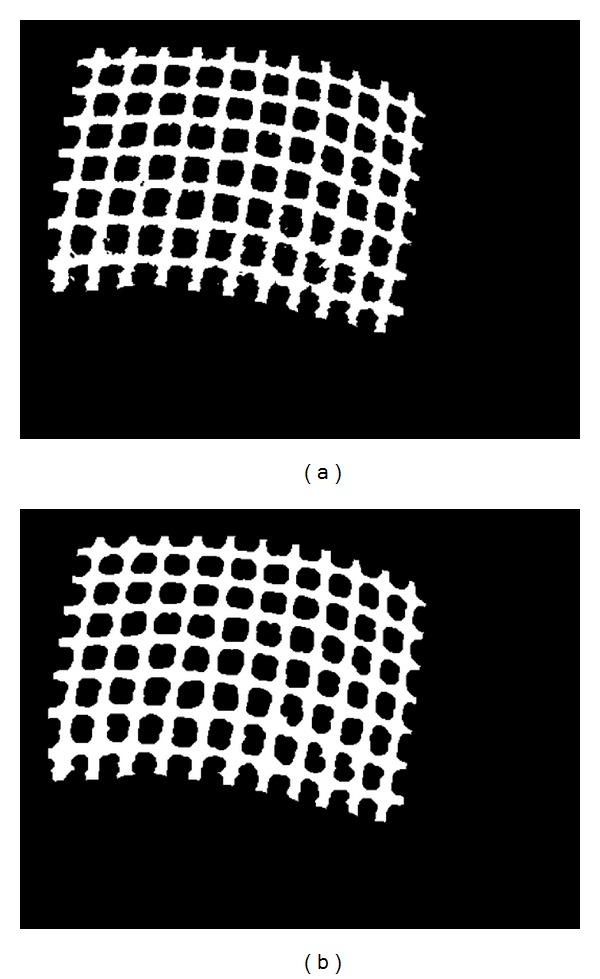
(a) Binary image before dilation and erosion (holes and spikes), (b) image after dilation and erosion process.

Because of the conversion to a binary image, some white pixels in the binary image are far away from the gridlines. Hence, these types of pixels could cause false positive pixels in the thinning process. By using dilation, we can expand the gridlines to fill the gaps between them and the protrusions pixels. Dilation process followed by erosion process is used to return the structure to its original state by removing the added structure of the gridlines. As a result, we have smoother gridlines without holes and protrusion pixels.  Figure 8 shows the binary images before and after dilation and erosion process, respectively.

### 3.5. Thinning and Intersection Detection

In order to detect the intersections of the gridline pattern, we used a thinning process applied to the above processed binary image. The thinning process generates an image with one pixel width; that is, it generates a skeleton image of the input binary image. Then we proceeded to detect the intersections of the image gridlines. This process was accomplished by applying a hybrid approach for cross-point detection called the combined cross-point number (CCN) method [6]. The CCN method uses two techniques to detect intersections of gridlines: simple cross-point number (SCN) and the modified cross-point number (MCN). The CCN algorithm is used to detect the intersection points of the gridlines.

In simple cross-point number, the image is iterated with a small window of size 3 by 3 pixels [6], as a result we have eight pixels surrounding the tested pixel. To test if the center pixel of the 3 by 3 window is a cross-point pixel, we iterate this window on the image and get the cross-point number (CPN) for the center pixel. The CPN is calculated by

(2) $$\begin{array}{c} {\text{CPN}}_{\text{SCN}}=\frac{1}{2}\overset{8}{\underset{n=1}{\sum }}\left|P_{n}-P_{n+1}\right|, \end{array}$$

where *P*_*n*_ is the pixel value of *n*th pixel of the 3 by 3 window and *P*_8_ = *P*_1_. A point is considered a cross point if its CPN is four.

**Figure 9:**
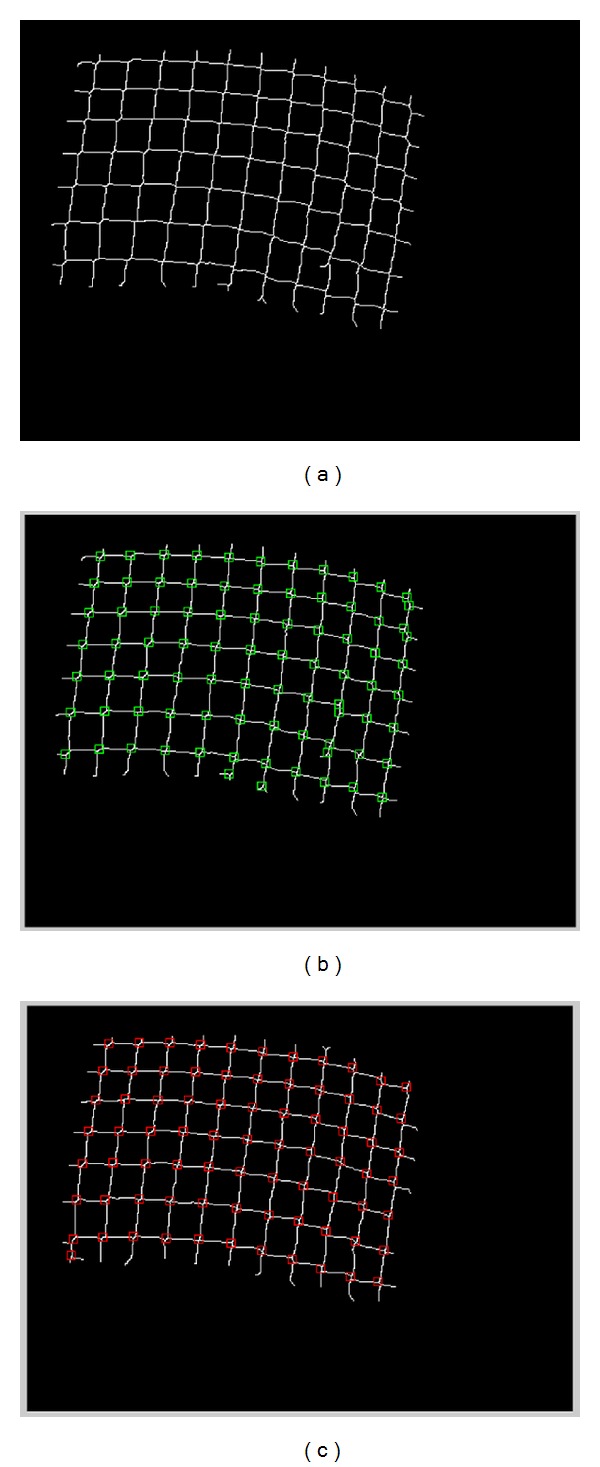
(a) An skeleton left image after thinning, (b) detected intersection points plotted on a left image, and (c) detected intersection points plotted on a right image.

**Figure 10:**
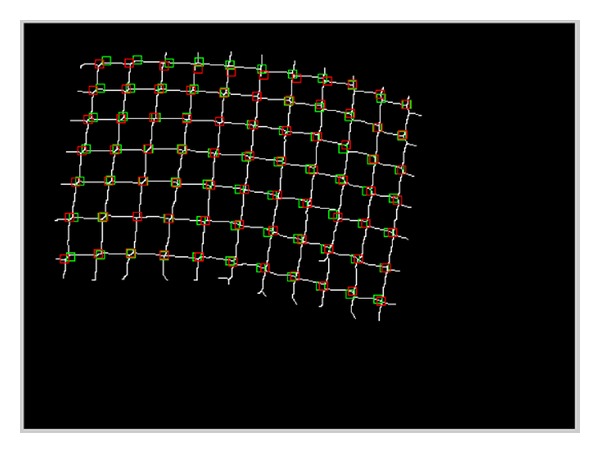
The matched points from both images.

In modified cross-point number method, the image is iterated by a window of size 5 by 5 pixels surrounding the center pixel. The CPN is calculated by

(3) $$\begin{array}{c} {\text{CPN}}_{\text{MCN}}=\frac{1}{2}\overset{16}{\underset{n=1}{\sum }}\left|P_{n}-P_{n+1}\right|, \end{array}$$

where *P*_17_ = *P*_1_. The pixel is considered a cross-point pixel if CPN_MCN_ ≥ 4. In the combined cross-point number both simple cross-point number and modified cross-point number methods are used. The simple cross-point number is used in the inner 3 × 3 neighbors of the center pixel, while the modified cross-point number is used in the outer 5 × 5 neighbors of the center pixel. Each pixel in the image has been tested against CPN using the modified cross-point number method, in which it is considered a cross point if and only if it satisfies CPN_SCN_ ≥ 4 and CPN_MCN_ ≥ 4. Because of the low quality of the images, we adjust the CPN of the combined CPN to be in the range of 3.0 and 4.0.  Figure 9(a) shows the left skeleton image by thinning operation.  Figure 9(b) shows the detected intersection points plotted on the left image.  Figure 9(c) shows the detected intersection points and plotted on the right image. Notice that there are false positive points in both images, and as shown in Figure 10, these false points are eliminated by our method using the epipolar geometry matching constraint.

### 3.6. Matching Grid Points

In order to reconstruct the surface within the ROI using the triangulation technique, we need to find the corresponding intersection points in the left and right images. Since these grid points have similar features, these correspondence relationships cannot be effectively obtained using conventional feature matching methods such as Scale Invariant Feature Transform (SIFT) and Speeded Up Robust Features (SURF) based techniques. In this paper, we adopt the method of epipolar constraints [2]. The intersection points are matched column-by-column to achieve good matching in our study. 

We have validated the proposed approach using 19 pairs of images acquired at different position and orientation of the stereo endoscope. Figure 10 shows the matched grid points superimposed in one image. Table 1 shows the actual number of points in each image, the number of the points detected, the number of correct points, the number of false positive points (FPP), and the number of false negative points (FNP). The average of sensitivity detection of the proposed method is 0.9822.

### 3.7. Points/Surface Reconstruction

**Figure 11:**
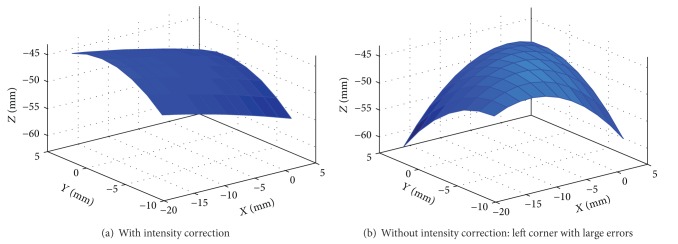
Reconstructed surface from stereo endoscopic images.

In general, a video image generated from the endoscope is a 2D projection of the 3D scene. This process can be represented using the pinhole camera model [8]. After we obtain the camera calibration parameters, we reconstruct a 3D point *x* from left and right image projections by using stereo triangulation. A smooth surface can be reconstructed by fitting these reconstructed 3D grid points as shown in Figure 11(a). This figure has demonstrated that the proposed method can achieve accurate surface reconstruction from the stereo endoscopic images.

In order to investigate the impact of the previous image processing procedures on the reconstructed surface, we have performed the following experiments. We use image intensity correction as an example to examine the effects in detail. We repeat the entire process of the surface reconstruction as shown in Figure 2, except that no intensity correction is performed.  Figure 11(b) shows the reconstructed surface without intensity correction, and Figure 14 shows the corresponding average registration error without intensity correction. Comparing Figures 11, 13, and 14, we can clearly see that intensity correction has significantly improved the reconstruction accuracy. With intensity correction, the average surface reconstruction error reduces from 1.86 mm to 0.76 mm. Similarly, the proposed automatic detection method for ROI improve the reconstruction accuracy as well. In Table 2, we show how the average surface error is affected by previous image processing procedures.

## 4. Surface Based Registration

### 4.1. ICP Registration

The Iterative Closest Point (ICP) algorithm is widely employed to align two three-dimensional surfaces. The ICP algorithm was first proposed by Besl and McKay [9], which is an iterative two-step method. The first step is to establish point correspondences by finding the corresponding point closest to the second surface for each point in the first surface. The second step is to calculate a transformation based on these matched points, which produces incremental transformations whose composition is the registration results.

**Figure 12:**
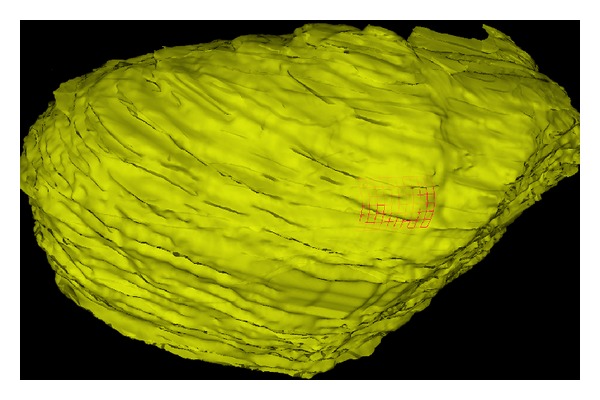
Overlay of 3D liver phantom surfaces after registration. Red mesh: reconstructed from endoscopic images, yellow surface: from MR images.

In this study, the ICP is employed to register the reconstructed surface from endoscopic images with the surface extracted from MR images.  Figure 12 shows the overlay of 3D surfaces after surface registration. 

### 4.2. Registration Accuracy

**Figure 13:**
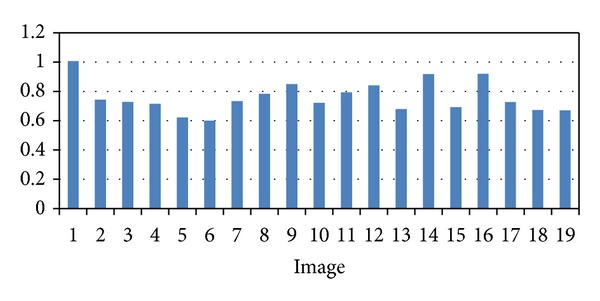
Surface registration accuracy wih intensity correction. Unit: mm.

**Figure 14:**
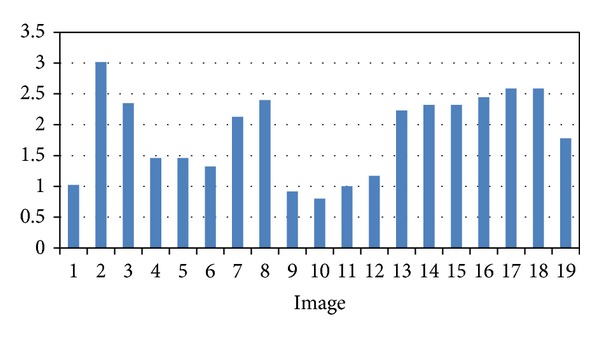
Surface registration accuracy without intensity correction. Unit: mm.

The projected gridline pattern used to test the proposed approach consists of seven rows and eleven columns, and we have 77 intersection points to detect in each image. We used 19 pairs of left and right images acquired by the stereo endoscope at different poses for validation. After ICP surface registration, we calculated the average surface distance (ASD) between two corresponding surfaces as registration accuracy. The resulting ASD is 0.76 ± 0.11 mm (see Figure 13). Figure 14 shows the corresponding registration accuracy without intensity correction.

**Figure 15:**
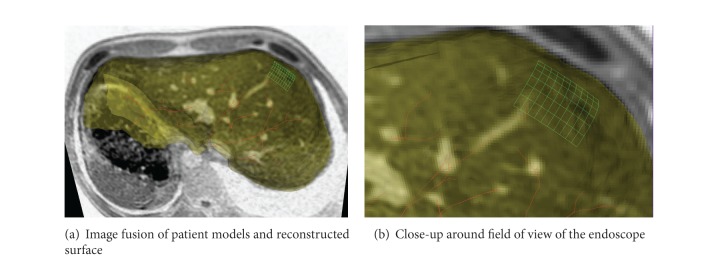
Fusion of patient models and reconstructed surface from the stereo endoscope. Green mesh is the reconstructed surface from two endoscopic images, red curves are the centerlines of vessels, segmented surface from MR images is shown in semitransparent yellow, and background is one MR slice with bright vessels.

After surface based registration, we are able to fuse the reconstructed surface from stereo endoscopic images with pre-operative high quality MR images and the corresponding patient-specific models such as vessel centerlines as shown in Figure 15. This will enable surgeons to see through critical structures beyond the operational site surface.

## 5. Discussion

Developing a rapid and accurate approach to reconstruct the surface from stereo endoscopic images is a very challenging task especially for soft tissues with few features. Many techniques have been developed to acquire or reconstruct surgical surface such as using laser scanners and Time-of-Flight (ToF) cameras [10–12]. Hayashibe et al. used a laser-scan endoscope technique to reconstruct the shape and texture of the area of interest [13]. A laser scanner was proposed to acquire the liver surface for image-guided liver surgery, but it took about 5–20 seconds [10]. Therefore it is not suitable for free-breathing patients since average respiratory rates of children are 16–30 breaths per minute. ToF cameras produce a depth map that can be immediately used to generate a 3D surface model in real time, but current devices are too large for endoscopic procedures [11, 12]. For this study, we employed a small stereo endoscope with the diameter of 4.9 mm, which can be used with a typical 5 mm trocar in clinical practice. For proof of concept, we used a general purpose projector to project the light pattern of gridlines. In the future, this easy-to-implement light pattern can be generated by using a very small lithographic pattern generator with 10 *μ*m thick lines at a distance of 50 *μ*m [14], which can generate a pattern of 50 × 50 lines within the size of 3 mm × 3 mm.

Many works [15–17], proposed to reconstruct the soft tissue structures of the abdomen using stereovision. However, it is difficult to find the correspondences between the two images, even when taking into account epipolar constraints. Moreover, 3D surface reconstruction for abdominal procedures is more challenging due to few or no features on the surface of some organs such as the liver. To tackle this, structured light based methods were presented in [18]. Traditionally, the structured light technique projects the coded pattern onto the object and substitutes one camera in the stereovision with a projector [19]. Consequently, the correspondence problem becomes a decoding problem, and we can determine the correspondences between the acquired image and the original known coded pattern. Many light patterns are proposed for 3D surface reconstruction in the last decades [20, 21]. The design and realization of a new endoscopic device by means of a robust structured light coding are presented in [4]. However, the coded pattern employed in [4] is not commercially available and is not easy to be implemented for clinical use.

Since we mainly consider minimally invasive image guided procedures, one major criterion of selecting light patterns is easy implementation. In this study, we select the gridline light pattern, which can be easily generated by a commercial projector or a special device. However, this choice of light pattern poses a great challenge for matching feature points (i.e., intersections) of gridlines due to symmetry and similar features of gridline points. Conventional methods [22, 23] such as SIFT cannot be employed effectively for surface reconstruction in our study. Based on the specific characteristics of the gridline light pattern in this paper, we adopt a new method for robust feature detection and matching.

In this study, we have proposed to use dedicated gridline light patterns to create noninvasive artificial features on the tissue surface, which is then used to robustly reconstruct 3D surface from stereo endoscopic images. This is a robust 3D surface reconstruction technique for procedures involving soft tissue organs especially with few surface features. Another feature of the proposed system is the use of a stereo endoscope for 3D surface reconstruction. The major advantage of using stereo endoscope is that it can acquire two synchronized images simultaneously, which provides necessary information for 3D surface reconstruction and eliminates the challenging temporal synchronization problem inherently with mono endoscope for moving deformable targets. The stereo endoscope not only provides two synchronized images at the same time but also reduces intra-operative image acquisition time and eliminates unnecessary motion of mono endoscope to acquire two images at different poses which is required for robust 3D surface reconstruction.

Effective and good display of virtual reality (VR) is an important factor for clinicians to accept and support the multimodality image guidance system in the clinical environment. It is still an active research topic as to how to present and fuse real images with patient-specific pre-operative images and models in an optimal way so that they register correctly in the physician's brain. Stereo display is one effective way to present the information to physicians. Some 3D video stereo monitors do not require a separate apparatus such as synch box, ZScreen, or active glasses. In order to effectively use our stereo endoscope, we still need 3D glasses in the current configuration; however, it would be more convenient for physicians to watch 3D video stereo monitor without glasses. Fused anatomy and models can provide physicians with more information where all supporting information becomes available. Multiple monitors can be used to display different information separately. For example, the first monitor can display real-time video stereo endoscopic images, while a second monitor is used to display the fused images/models. Thus in challenging scenarios, doctors can selectively watch different monitors to acquire needed information including surgical plans and other critical structures such as tumors and blood vessels without interference from unnecessary information. 

## 6. Conclusion

In this study, we proposed a novel approach to match stereo endoscopic images and MR images. The proposed surface-based registration has proved to be an effective method for registering these images of different imaging mechanisms. Moreover, the light patterns of the gridlines facilitated the surface reconstruction of surgical sites with few surface features. In this paper, we validated the proposed method with static objects; however, our method has the potential to be extended to procedures involving moving organs. 

We have demonstrated the effectiveness of our technique in registration of the reconstructed surface with the surface extracted from MR images of a liver phantom. We have shown that various image processing techniques we used before the image registration have a significant impact on the resulting registration accuracy. We have achieved a surface registration accuracy of 0.76 ± 0.11 mm. The proposed technique has the potential to be used in clinical practice to improve image guidance in endoscope based minimally invasive procedures. The fused image guidance may also be applied to the endoscopic procedures of other organs in the abdomen, chest cavity, and pelvis such as the kidneys and the lungs.

Future work includes integration of our method into the clinical image guidance system and further validation by animal study and clinical study.

## Figures and Tables

**Table:** (a) With intensity correction

Image	Number of points	Detected points	Correct points	Sensitivity	FPP	FNP
Im1	77	73	73	0.9481	0	4
Im2	77	74	74	0.961	0	3
Im3	77	74	74	0.961	0	3
Im4	77	73	73	0.9481	0	4
Im5	77	76	76	0.987	0	1
Im6	77	78	77	1.0	1	0
Im7	77	77	76	0.987	1	1
Im8	77	76	76	0.987	0	1
Im9	77	76	76	0.987	0	1
Im10	77	76	76	0.987	0	1
Im11	77	76	76	0.987	0	1
Im12	77	76	76	0.987	0	1
Im13	77	77	77	1.0	0	0
Im14	77	76	76	0.987	0	1
Im15	77	77	77	1.0	0	0
Im16	77	76	76	0.987	0	1
Im17	77	76	76	0.987	0	1
Im18	77	76	76	0.987	0	1
Im19	77	76	76	0.987	0	1

Average				0.9822		

**Table:** (b) Without intensity correction

Image	Number of points	Detected points	Correct points	Sensitivity	FPP	FNP
Im1	77	51	51	0.66	0	26
Im2	77	48	48	0.62	0	29
Im3	77	25	24	0.31	1	53
Im4	77	53	52	0.67	1	25
Im5	77	53	53	0.68	0	24
Im6	77	39	38	0.49	1	39
Im7	77	42	42	0.54	0	35
Im8	77	39	39	0.50	0	38
Im9	77	60	60	0.77	0	17
Im10	77	56	55	0.71	1	22
Im11	77	53	53	0.68	0	24
Im12	77	52	52	0.67	0	25
Im13	77	44	44	0.57	0	33
Im14	77	42	41	0.53	1	36
Im15	77	42	40	0.51	2	37
Im16	77	41	40	0.51	1	37
Im17	77	36	35	0.45	1	42
Im18	77	36	35	0.45	1	42
Im19	77	41	33	0.42	2	44

Average				0.5653		

**Table 2:** Average surface distance error (ASD) impacted by image processing procedures.

Case	Mean (mm)	Standard deviation (mm)
Proposed method	0.76	0.11
Without intensity correction	1.86	0.68
Without detection of ROI	1.24	0.73
